# Round the Hospitals

**Published:** 1917-04-28

**Authors:** 


### ROUND THE HOSPITALS.

The Nurses' Concert to be held at the Queen's Hall on Wednesday, May 2, promises to be one of the events of the season. Queen Alexandra has intimated her intention of being present to meet her nurses, thousands of whom have already joined the College Register, the success of which is now assured. The Concert will be the opening day of the campaign for the Endowment Fund of the Royal College of British Nursing which two powerful organisations of women, who have already done splendid monetary service for the Red Cross, have generously undertaken to raise with as little delay as possible. Little has been said lately of the Royal College of British Nursing, but that is Mr. Stanley's way. Wise man that he is, he first gets his machinery together and in working order, and then takes the public into his confidence with the co-operation and powerful aid of the newspaper press. In four months two thousand certificated nurses have sent in their applications to the Editor of *The Nursing Mirror* for Registration with the College. The number of nurses applying for Registration with the College, immediately the Endowment Fund is launched and the money continues to flow in, should so increase, as to make it a race between the ladies who are raising the Endowment Fund and the certificated nurses who hasten to become members of the Royal College of British Nursing. This race for victory will demonstrate whether British certificated nurses will complete the College Register first by applying for Registration now, or British women will complete the Endowment Fund of the College. This race will interest the whole nation and Empire. We anticipate that every certificated nurse will make it her immediate business to place her name on the College Register to-day, for then Statutory Recognition, Registration, Status and Protection, with assured certainty, will be quickly theirs.

---

Miss Sordy, matron of Queen Mary's Hospital for the East End, Stratford, E., is to be congratulated on the large attendance and complete success of the meeting in support of the Royal College of British Nursing, which she organised at her hospital on April 18. The chairman of the hospital, Mr. C. E. Leo Lyle, presided. The matrons of all the local hospitals, infirmaries, and nursing homes were present, including Miss L. Clark, matron of West Ham Infirmary; Miss Forsyth, matron of the Isolation Hospital, Shadwell; Miss Green, matron of the Ilford General Hospital; Miss Foster, matron of the Walthamstow Hospital; and Miss Trewin, matron of the Nursing Home, Whipp's Cross Road. Many sisters and nurses also attended, as well as nurses attached to schools under the control of the London County and other Councils. A summary of Mr. Stanley's address is given on p. 73.

---

On March 17 we published the names of the then members of the Irish Board of the Royal College of British Nursing. Since that date three members have been added, viz., Miss Brand, of Queen Victoria's Jubilee Institute for Nurses; Sir Alexander Dempsey, Belfast; and Mr. Wilfred FitzGerald. The following have been appointed honorary officers to the Irish Board: Dr. George Peacocke, Chairman; Sir Andrew Horne, Vice-Chairman; Mr. Andrew Beatty, D.L., Treasurer; Miss Reed, Honorary Secretary. The six delegates nominated by this Board to act on the Council in London are the Chairman, the Vice-Chairman, the Hon. Treasurer, the Hon. Secretary, Miss Eddison, and Miss Curtin. Miss Matheson, daughter of Mr. Charles L. Matheson, K.C., and niece of the Right Hon. Sir Robert Matheson, has been appointed Secretary. Miss Matheson, who is a trained nurse, is a distinguished graduate of Dublin University. Excellent offices have been opened in Kildare Street, Dublin. No propaganda has yet been considered, but, now that the Board is complete and the machinery in order, work will soon begin. It would be as yet premature to discuss what schemes are in contemplation, but they include the raising of the status of Irish nurses and the betterment of their conditions of service, which will comprehend some form of Nurses' Benevolent Society.

---

It is interesting to mark how closely the tactics of the British nursing all-highest resemble the German model. She is responsible for the policy of the "Smash the College" and "Divide the Nurses" party, the members of which have found shelter under the Lady's umbrella, presented to the public and the nursing profession as the Central Committee for the State Registration of Nurses. The Lady's "Wireless" last week substituted anonymity for the signatures of the all-highest or that of one of the three feminine echoes which have ended previous manifestoes. Have the faithful echoes found their souls, refused their name to the twenty-year-old form of personal abuse and name-slinging, and so compelled a return to anonymity? The latest attack rests upon the assumption that a knowledge of the facts is the sole prerogative of the Lady, for how otherwise could she venture to attack members of the College Council by propounding one question in regard to training certificates which she, out of fulness of knowledge, might possibly have applied to herself? As a matter of fact, however such a question may affect the Lady, it has no relevancy at all, we are informed, when applied to each and

**ROUND THE HOSPITALS—(*continued*).**

all of the present members of the Council of the Royal College of British Nursing.

---

*Quem Jupiter vult perdere prius dementat.* In The Hospital of March 24, page 501, in dealing with the problem of Poor-Law infirmaries, one of our workers wrote of the workhouse influence. Speaking of the paramount importance of separating infirmaries from the workhouse, one of the ablest and most experienced Poor-Law nursing authorities wrote: "The influence and authority of the workhouse is fatal to really good training unless it is continually kept at bay. . . . Nurses under the workhouse influence tend to become rough, contemptuous, and rude in manner to their patients, exactly as the average workhouse officer is to the 'inmates' under his charge." These are demonstrable truths known to everyone familiar with Poor-Law conditions, including the Editor of the *Journal* and, presumably, of all practical members of the National Poor-Law Officers' Association. The habit of treating British nurses as noodles and "know-nothings" is demonstrably dangerous to the reputation of the slanderer. That danger is apt to increase when the slanderer's object is to enlist the support, for political reasons, of an organisation like the National Poor-Law Officers' Association. Could more convincing proof of this be produced than the following reprint from page 178 of the *Journal* of the 21st instant?

> *The Poor-Law Officers' Journal* is protesting in no uncertain terms against Sir Henry Burdett's insulting attack in his Hospital newspaper on Poor-Law Nurses as a class—in which publication they were recently described as of "rough, contemptuous, and rude conduct" towards their patients. It is a wholesale insult to a most self-sacrificing body of women which could emanate from no other source, Poor-Law nurses are venturing to inquire into the College constitution, and some of them have had the daring to object to it. Thus this thusness. Our advice to Poor-Law nurses is to stand up to every form of intimidation—journalistic or otherwise. We [the *Journal*] congratulate the *Poor-Law Officers' Journal* on setting them the example.

It will be observed that our contributor's accurate description that nurses under the workhouse influence tend to become "rough, contemptuous, and rude in manner to their patients" is stated to apply not to the nurses specifically defined by our contributor, but to "Poor-Law nurses as a class." And on this shameful travesty is hung a flagrant misrepresentation of facts and a wanton attempt to prejudice their readers against The Hospital by attributing to it views and statements the exact opposite to those expressed in its columns. It would be difficult to find a paragraph anywhere in the whole British Press so flagrant, wanton, and discreditable to those responsible for it as the one we here quote. We take it that such a descent from decency to the dust-heap is an intimation of the conviction of those responsible that they represent a losing cause so far as Poor-Law certificated nurses and British nurses generally are concerned.

For "Unveiling of Bas-relief of Florence Nightingale," see p. 66.
For "The Royal College of British Nursing: Speech by the Hon. Arthur Stanley," see p. 73.

